# The Tubercle Parasite—A New Theory of Consumption

**Published:** 1882-06

**Authors:** 


### SELECTIONS.

### THE TUBERCLE PARASITE.—A NEW THEORY OF CONSUMPTION.

Prof. John Tyndall gives the following luminous account of Dr. Koch's investigations in a letter to the London *Times*:

On the 24th of March, 1882, an address of very serious public import was delivered by Dr. Koch before the Physiological Society of Berlin. It touches a question in which we are all at present interested—that of experimental physiology—and I may, therefore, be permitted to give some account of it in *The Times.* The address, a copy of which has been courteously sent to me by its author, is entitled "The Etiology of Tubercular Disease." Koch first made himself known by the penetration, skill, and thoroughness of his researches on the contagion of splenic fever. By a process of inoculation and infection he traced this terrible parasite through all its stages of development and through its various modes of action. This masterly investigation caused the young physician to be transferred from a modest country practice, in the neighborhood of Breslau, to the post of Government Advisor in the Imperial Health Department of Berlin.

From this department has lately issued a most important series of investigations on the etiology of infective disorders. Koch's last inquiry deals with a disease, which, in point of mortality, stands at the head of them all. If, he says, the seriousness of a malady be measured by the number of its victims, then the most dreaded pests which have hitherto ravaged the world—plague and cholera included—must stand far behind the one now under consideration. Koch makes the startling statement that one-seventh of the deaths of the human race are due to tubercular disease, while fully one-third of those who die in active middle-age are carried off by the same cause. Prior to Koch it had been placed beyond doubt that the disease was communicable; and the aim of the Berlin physician has been to determine the precise character of the contagium which previous experiments on inoculation and inhalation had proved to be capable of indefinite transfer and reproduction. He subjected the diseased organs of a great number of men and animals to microscopic examination, and found, in all cases, the tubercles infested with a minute, rod-shaped parasite, which by means of a special dye, he differentiated from the surrounding tissue. It was, he says, in the highest degree impressive to observe in the centre of the tubercle cell the minute organism which had created it. Transferring directly, by inoculation, the tuberculous matter from diseased animals to healthy ones, he in every instance reproduced the disease. To meet the objection that it was not the parasite itself, but some virus in which it was imbedded in the diseased organ, that was the real contagium, he cultivated his bacilli artificially, for long periods of time and through many successive generations. With a speck of matter, for example, from a tuberculous human lung, he infected a substance prepared, after much trial, by himself, with a view of affording nutriment to the parasite. Here he permitted it to grow and multiply. From this new generation he took a minute sample and infected therewith fresh nutritive matter, thus producing another brood. Generation after generation of bacilli were developed in this way, without the intervention of disease. At the end of the process, which sometimes embraced successive cultivations extending over half a year, the purified bacilli were introduced into the circulation of healthy animals of various kinds. In every case inoculation was followed by the reproduction of the parasite and the generation of the original disease.

Permit me to give a further, though still brief and sketchy, account of Koch's experiments. Of six guinea-pigs, all in good health, four were inoculated with bacilli derived originally from a human lung, which in fifty-four days had produced five successive generations. Two of the six animals were not infected. In every one of the infected cases the guinea-pig sickened and lost flesh. After thirty two days one of them died, and after thirty-five days the remaining five were killed and examined. In the guinea-pig that died, and in the three remaining infected ones, strongly pronounced tubercular disease had set in. Spleen, liver, and lungs were found filled with tubercles; while in the two uninfected animals no trace of the disease was observed. In a second experiment, six out of eight guinea-pigs were inoculated with cultivated bacilli, derived originally from the tuberculous lung of a monkey, bred and rebred for ninety-five days, until eight generations had been produced. Every one of these animals was attacked, while the two uninfected guinea-pigs remained perfectly healthy. Similar experiments were made with cats, rabbits, rats, mice, and other animals, and without exception it was found that the injection of the parasite into the animal system was followed by decided and, in most cases, virulent tubercular disease.

In the cases thus far mentioned inoculation had been effected in the abdomen. The place of inoculation was afterward changed to the aqueous humor of the eye. Three rabbits received each a speck of bacillus-culture, derived originally from a human lung affected with pneumonia. Eighty-nine days had been devoted to the culture of the organism. The infected rabbits rapidly lost flesh, and after twenty-five days were killed and examined. The lungs of every one of them were found charged with tubercles. Of three other rabbits, one received an injection of pure blood-serum in the aqueous humor of the eye, while the other two were infected in a similar way, with the same serum, containing bacilli derived originally from a diseased lung, and subjected to ninety-one days' cultivation. After twenty-eight days the rabbits were killed. The one which had received an injection of pure serum was found perfectly healthy, while the lungs of the two others were found overspread with tubercles.

Other experiments are recorded in this admirable essay, from which the weightiest practical conclusions can be drawn. Koch determines the limit of temperature between which the tubercle-bacillus can develop and multiply. The minimum temperature he finds to be 86° Fahrenheit and the maximum 140°. He concludes that, unlike the bacillus anthracis of splenic fever, which can flourish freely outside the animal body, in the temperate zone animal warmth is necessary for the propagation of the newly discovered organism. In a vast number of cases Koch has examined the matter expectorated from the lungs of persons affected with phthisis, and found in it swarms of bacilli, while in matter expectorated from the lungs of persons not thus afflicted, he has never found the organism. The expectorated matter in the former cases was highly infective, nor did drying destroy its virulence. Guinea-pigs infected with expectorated matter which had been kept dry for two, four, and eight weeks respectively, were smitten with tubercular disease quite as virulent as that produced by fresh expectoration.

Koch points to the grave danger of inhaling air in which particles of the dried sputa of consumptive patients mingles with dust of other kinds.

It would be mere impertinence on my part to draw the obvious moral from these experiments. In no other conceivable way than that pursued by Koch could the true character of the most destructive malady by which humanity is now assailed be determined. And, however noisy the fanaticism of the moment may be, the common sense of Englishmen will not, in the long run, permit it to enact cruelty in the name of tenderness, or to debar us from the light and leading of such investigations as that which is here so imperfectly described.

Your obedient servant,

HIND HEAD, April 20. JOHN TYNDALL.

